# Impact on the Thoracic and Cardiovascular Surgery Residents’ Learning Curve During the COVID-19 Pandemic

**DOI:** 10.21470/1678-9741-2020-0300

**Published:** 2020

**Authors:** Wildor Samir Cubas Llalle, David Bellido-Yarlequé, Cristian Yépez-Calderón, Priscilla Chávarry-Infante

**Affiliations:** 1 Department of Thoracic and Cardiovascular Surgery, Edgardo Rebagliati Martins National Hospital, Lima, Peru.; 2 Department of Thoracic and Cardiovascular Surgery, Guillermo Almenara Irigoyen National Hospital, Lima, Peru.; 3 Department of Cardiovascular Surgery, Carlos Alberto Peschiera Carrillo National Cardiovascular Institute, Lima, Peru.; 4 Department of Thoracic and Cardiovascular Surgery, Hipólito Unánue National Hospital, Lima, Peru.

**Dear Editor,**

The coronavirus disease (COVID-19) pandemic has been drastically challenging the integrity of health systems in more than 100 countries around the world (>6,000,000 infected; >370,000 deaths), including Peru, where it has been considered by many experts as the new epicenter of the pandemic in South America (9^th^ worldwide, >5,500 cases/1,000,000 people), due to the exponential growth of infection (>180,000 people) and mortality (>5,000 people) in the general population as well as in health personnel (>150 deaths).

Since March 15, 2020, the date on which the state of sanitary emergency was decreed in our country (>80 days of social isolation so far), the Ministry of Health, following the guidelines proposed by several international organizations^[1]^, developed an unprecedented series of public health policies to reduce the infection curve and mortality rates attributed to COVID-19. Many of these measures were the optimization of medical care for those infected with the implementation of a differentiated triage, a greater number of beds in intensive care units with support for mechanical ventilation and the displacement of all health personnel to critical care areas for COVID-19 patients; all of this was developed at the cost of suspending and cancelling "non-essential" medical services that include elective surgical procedures.

**Fig. 1:**
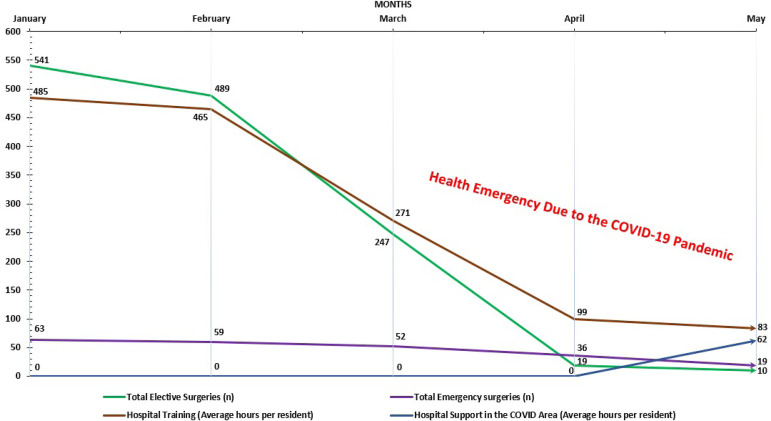
Impact in the thoracic and cardiovascular surgery residency in the main Peruvian hospitals during the COVID-19 pandemic.

This last provision indirectly affected the learning curve of residents of the surgical area, as is the case with thoracic and cardiovascular surgery, evidencing a reduction in surgical procedures (elective *vs*. emergency) and causing a decrease in the hours of surgical training that were progressively replaced by support hours in specific areas for COVID-19 (Figure 1).

The success of the learning curve in thoracic and cardiovascular surgery residents is determined by the circle of permanent and uninterrupted learning, articulated by 3 fundamental pillars: the theoretical component consisting mainly of the constant review of the medical literature and the periodic participation in clinical-surgical conferences or gatherings led by mentor surgeons; the component of strengthening operational skills that allow the acquisition of procedural skills through training using simulation; and, finally, the practical component, in which the resident actively participates as an observer, assistant or main surgeon in various surgical procedures. However, all this learning process has been considerably affected due to the COVID-19 pandemic, since many components of the process were reduced to a nullity to minimize the coronavirus exposure and contagion.

Likewise, due to the crisis that our health system is going through, as a consequence of the deficit in infrastructure and medical personnel due to the high demand of infected patients, several departments of thoracic and cardiovascular surgery in the country have arranged to provide support, with their surgeons and residents, along with the entire hospital environment for COVID care, in order to provide assistance in the face of the impending hospital collapse.

**Fig. 2:**
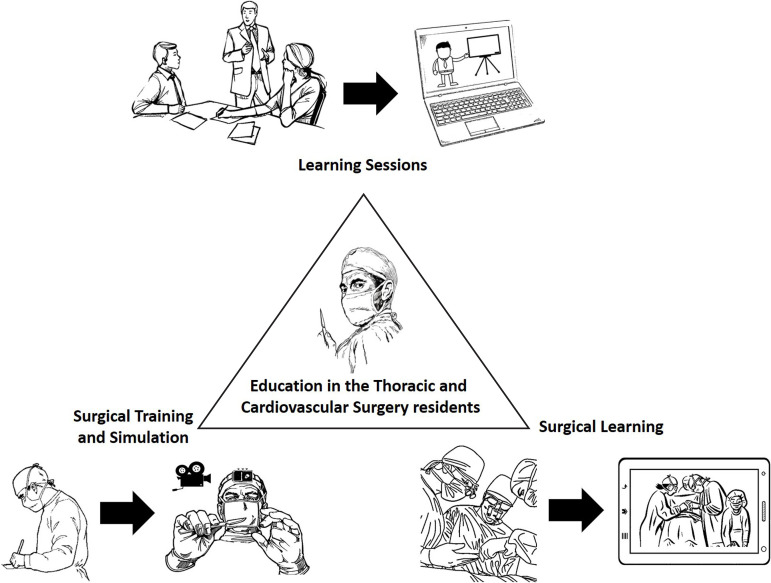
Adaptation of the three fundamental pillars of surgical education in residents of thoracic and cardiovascular surgery. The clinical-surgical sessions and conferences would be adapted to videoconferences and/or webinars, training and simulation of procedural skills could be replaced by recording them under live supervision by a mentor surgeon and the practical component that includes surgical procedures could be recorded for later viewing.

Given the great role that the COVID-19 pandemic has been generating and its undeniable negative impact on the learning curve in the residency program in thoracic and cardiovascular surgery, a variety of technological alternatives have emerged to try to compensate the lack of traditional academic training in medical residency towards a virtual hybrid model^[2]^. Softwares and platforms such as Zoom, GoToMeeting, Google Meet, Skype and others have allowed to resume some academic activities (videoconferences, webinars, training recording and/or simulation of operative skills and online transmission of surgical procedures) from an “e-learning” perspective (Figure 2)^[3,4]^.

Among the main benefits described on the use of these learning platforms, stand out their versatility and ease of use, allowing interaction, discussion and/or live debate between the participants, generating an optimal learning environment; in addition, they can be recorded with the aim of viewing them again and/or sharing them with an audience of interest (video-based education - VBE). In Peru, this unusual way of learning has been gaining great expectations and acceptance in the community of attending physicians and residents of thoracic and cardiovascular surgery. However, this educational model has been replicated long before in countries such as Italy and the United States, incorporating it into the residency training system and obtaining a great impact on the learning curve^[3,5]^.

Some organizations in the world related to our surgical field have been providing a series of surgical learning platforms free of charge by highly prestigious mentor surgeons, to strengthen and contribute to continuing education among their members and the public of interest; this is the case of the Society of Thoracic Surgeons (STS, https://www.sts.org/learning-center/webinars), European Association for Cardio-Thoracic Surgery (EACTS, http://www.eacts.org/education/ests_school/webinars.aspx) and Houston Methodist DeBakey Heart & Vascular Center (https://www.youtube.com/channel/UCb8PGmJ6SILfyOvOWJvHZIg).

Given the imminent negative impact on the learning curve of thoracic and cardiovascular surgery residents due to the COVID-19 pandemic, there is an urgent need to recondition a new multifaceted approach that allows for virtual surgical education. The new and unusual hospital environment caused by the health emergency has provided a great opportunity to develop novel learning strategies in the various departments of our specialty, using several online platforms that make it possible to dispense with geospatial and temporal limitations. As our surgical field joins the lines of care to face this unprecedented global crisis, various medical residency programs around the world are promoting the transition from a traditional learning model to a technological and intelligent one, translating into an important milestone in training future residents.
